# The impact of cardiovascular disease on all-cause and cancer mortality: results from a 16-year follow-up of a German breast cancer case–control study

**DOI:** 10.1186/s13058-023-01680-x

**Published:** 2023-07-27

**Authors:** Annika Möhl, Sabine Behrens, Fabian Flaßkamp, Nadia Obi, Annika Kreienbrinck, Bernd Holleczek, Kathleen Gali, Jenny Chang-Claude, Heiko Becher

**Affiliations:** 1 Institute of Medical Biometry and Epidemiology, University Medical Center Hamburg-Eppendorf, Martinistraße 52, 20246 Hamburg, Germany; 2 Division of Cancer Epidemiology, German Cancer Research Centre, Im Neuenheimer Feld 280, 69120 Heidelberg, Germany; 3 Department of Medical Psychology, University Medical Center Hamburg-Eppendorf, Martinistraße 52, 20246 Hamburg, Germany; 4 Saarland Cancer Registry, Neugeländstraße 9, 66117 Saarbrücken, Germany; 5 Hamburg Center for Health Economics, University of Hamburg, Esplanade 36, 20354 Hamburg, Germany; 6 Cancer Epidemiology Group, University Cancer Center Hamburg, University Medical Center Hamburg-Eppendorf, Martinistraße 52, 20246 Hamburg, Germany; 7 Institute of Global Health, University Hospital Heidelberg, Im Neuenheimer Feld 130.3, 69120 Heidelberg, Germany

**Keywords:** Breast cancer, Mortality, Cardiovascular disease, Case–control study

## Abstract

**Background:**

Cardiovascular disease (CVD) is the leading cause of death worldwide. The aim of this study was to examine if CVD affects the mortality of women after a breast cancer diagnosis and population controls differently.

**Methods:**

The analysis included a total of 3,555 women, diagnosed with primary stage 1–3 breast cancer or in situ carcinoma between 2002 and 2005 and 7,334 controls breast cancer-free at recruitment, all aged 50–74 years, who were followed-up in a German breast cancer case–control study until June, 30 2020. Kaplan–Meier and cumulative incidence function were calculated for all-cause mortality and mortality from any cancer, stratified for case–control status and CVD, separately for women aged < 65 and ≥ 65 years. Cox regression and Fine-Gray subdistribution hazard models were used to estimate hazard ratios (HR) and 95% confidence intervals (95% CI) for the association between case–control-status, CVD and mortality from all causes/any cancer.

**Results:**

The median follow-up was 16.1 years. In total, 1,172 cases (33.0%) and 1,401 initial controls (19.1%) died. CVD prevalence at recruitment was 15.2% in cases and controls. Cases with CVD had the highest and controls without CVD the lowest mortality during the entire observation period in both age groups (< 65 and ≥ 65 years). CVD was identified as a risk factor for all-cause mortality in both cases and controls aged < 65 years (HR 1.22, 95%CI 0.96–1.55 and HR 1.79, 95%CI 1.43–2.24) as well as at ages of ≥ 65 years (HR 1.44, 95%CI 1.20–1.73 and HR 1.59, 95%CI 1.37–1.83). A significant association of CVD and cancer mortality was found only for cases aged ≥ 65 years.

**Conclusion:**

CVD was significantly associated with all-cause mortality of both cases and controls and CVD was identified as a risk factor for cancer mortality of cases aged ≥ 65 years at recruitment. Therefore, attention should be paid on monitoring and preventing CVD in breast cancer patients, especially in those diagnosed at older ages.

**Supplementary Information:**

The online version contains supplementary material available at 10.1186/s13058-023-01680-x.

## Introduction

In 2020, 2.26 million new diagnoses of breast cancer and 685,000 deaths were observed worldwide. Breast cancer is among the top five cancer-related causes of death [1] but breast cancer mortality in European Countries declined during the last decades, especially in the Northern and Western countries [2, 3]. Improvement in treatments, diagnosis and disease management as well as systematic screening programs have contributed towards the reduction in mortality [2, 4–7]. In the growing group of aging breast cancer survivors, other comorbidities, especially cardiovascular diseases (CVD), are of great importance [8, 9].

Breast cancer patients with prevalent comorbidities, including CVD, have poorer survival outcomes than patients without comorbidities [10]. In a large U.S. breast cancer cohort (n = 63,566), breast cancer patients with CVD had a 1.24-fold higher risk of dying from breast cancer compared to patients without CVD [11]. As a consequence of cardiotoxicity, breast cancer therapies such as radiotherapy can promote incident cardiac events in the short and long-term [12]. Shared risk factors of breast cancer and cardiovascular disease, such as lifestyle factors including tobacco or alcohol use, obesity and a lack of physical activity may worsen cardiotoxicity of cancer treatments [13].

In 2018, the American Heart Association published a scientific statement on CVD and breast cancer to give an overview of the intersection of both diseases including shared risk factors and cardiotoxicity of treatments. The authors pointed out that in older women CVD represents a greater risk for mortality than the cancer diagnosis itself [14].

Ten years after diagnosis, the probability of dying from other causes than breast cancer, of which heart diseases were most common (1,727 of 7,271 deaths), was 0.20 whereas the probability of dying from breast cancer was only 0.04 [15]. Furthermore, two case–control studies of Bradshaw et al. and Ramin et al. showed that survivors of breast cancer had a higher risk of dying from CVD more than 7 and 8 years after diagnosis, respectively, compared to women without breast cancer [16, 17]. A systematic review from 2017, including 14 studies of different designs, also showed a higher CVD mortality among women with breast cancer compared to the general population [18].

Although many studies compared CVD mortality in breast cancer survivors and controls, there is a lack of studies investigating the impact of CVD on mortality, especially with focussing on the difference between women after breast cancer diagnosis and population controls.

The aim of this study was to investigate whether cardiovascular diseases (CVD) affect all-cause mortality and cancer-specific mortality differently in women after a breast cancer diagnosis (cases) and controls without a breast cancer diagnosis from the German MARIE-study (Mamma Carcinoma Risk Factor Investigation).

## Materials and methods

### Study population and design

We used data from 11,154 women enrolled in the prospective population-based case–control study MARIE (Mamma Carcinoma Risk Factor Investigation) which aimed to investigate risk factors for breast cancer. Details of the study design have been described elsewhere [19]. In short, cases aged 50 to 74 years (n = 3,813) who were diagnosed with histologically confirmed stage 1 to 4 primary invasive breast cancer or carcinoma in situ between 2001 and 2005 were extracted from hospitals and pathology records in Hamburg and the region Rhine-Neckar-Karlsruhe and from the cancer registry Hamburg. Population-based age- and region-matched controls were selected from registration offices, of which 7,341 completed the baseline interview. Patients diagnosed with stage 4 breast cancer, pre-OP chemo (n = 255) or missing stage (n = 2), controls diagnosed with breast cancer before 2006 (n = 6) as well as women with missing information on CVD at baseline (n = 2) were excluded from the analyses, resulting in 3,555 cases and 7,334 controls for this analysis.

### Assessment of cardiovascular disease and covariates

Information on covariates was obtained at a standardized face-to-face interview at recruitment.The participants were asked if they have ever been diagnosed with any of the comorbidities listed in the questionnaire. Other diseases could be specified via free text fields. In our analysis, the binary exposure variable CVD was defined as having ever been diagnosed with at least one of the following diseases until recruitment: myocardial infarction, congestive heart failure, peripheral vascular disease, angina pectoris, arrhythmia or stroke.

### Assessment of vital status and causes of death

Vital status (alive, dead, lost to follow-up) and exact dates of death and lost to follow-up were obtained from the German registration offices for every woman on 30th June 2020. Death certificates were requested from the local health offices for the entire observation period. Causes of death were coded according to the 10th revision of the International Statistical Classification of Diseases and Related Health Problems (ICD-10 WHO).

### Statistical analyses

The observation period was from the date of the baseline interview until death, last date known to be alive or the 30th of June 2020, which ever came first. Following descriptive analyses for relevant variables, Kaplan–Meier survival curves for all-cause mortality and cumulative incidence functions for mortality of any cancer (referred to as "cancer mortality") were estimated, stratified for case–control status and CVD and separately for women aged < 65 years and ≥ 65 years at recruitment. Median follow-up time was calculated using reverse Kaplan–Meier method.

Cox regression models were used to estimate hazard ratios (HR) with 95% confidence intervals (95% CIs) for the association of CVD at baseline, including the interaction with case–control status, and all-cause mortality. Due to the known interaction between cancer (treatments) and CVD [20], cancer mortality was investigated separately using Cox regression (cause-specific HRs) and Fine-Gray models (subdistribution HRs). Age was used as the time-scale (start: age at baseline, end: age at date of death or censoring).

As mentioned above, two separate models were calculated for the age groups < 65 years and ≥ 65 years, since the population of people aged ≥ 65 will rapidly grow in the next decades and the CVD burden will increase [21]. Age is one of the most significant factors influencing survival of breast cancer patients in general as well as the risk of CVD and dying from CVD [9, 18].

Covariates to be entered in the models were chosen based on hypotheses concerning their relationship with CVD and mortality, using directed acyclic graphs (DAG) [22]. Based on the DAG (Additional file 1: Figure S1) we adjusted for the following baseline variables: body mass index (BMI, continuous), education (low, middle, high), smoking status (never, former, current), alcohol consumption (abstinent, < 19 g/day, ≥ 19 g/day), physical activity since age of 50 years based on walking, cycling and recreational physical activity (MET hours/week, continuous; if information was missing and women were aged < 56, information about physical activity since age of 30 years was used), diabetes, cancer other than breast cancer prior to baseline and living with a partner (yes/no).

For supplemental analysis, CVD mortality was compared between cases and controls using Cox regression (cause-specific) and Fine-Gray models, stratified by age groups (< 65 years and ≥ 65 years) and adjusted for the above mentioned covariates.

All analyses were performed using SAS Software, version 9.4 of the SAS system for Windows (Copyright © 2002–2012 SAS Institute Inc., Cary, NC, USA).

## Results

### Characteristics of the study population

A total of 10,889 women (3,555 cases, 7,334 controls) were included in the analyses. Overall, case and control characteristics were comparable, but there were differences between women aged < 65 years (n = 6,480) and those ≥ 65 years (n = 4,409) at baseline.

The BMI at baseline was slightly higher in the elderly. The proportions of women having a low educational level, being never smokers and being abstinent were higher in those aged ≥ 65 years than in the younger women but similar in cases and controls. While only 11.8% (cases) and 11.4% (controls) of the older women were current smokers, 25.2% (cases) and 25.3% (controls) of the younger women were current smokers. Physical activity was about 50 METs per week in cases and controls in both age groups (Table 1).

**Table 1:** Characteristics of the study population at baseline, stratified for age groups and case–control status, n = 10,889

	< 65 years		≥ 65 years	
	Cases (%) n = 2123	Controls (%) n = 4357	Cases (%) n = 1432	Controls (%) n = 2977
*Study region (missing* = *0)*				
Hamburg	1175 (55.3%)	2358 (54.1%)	825 (57.6%)	1644 (55.2%)
Rhine-Neckar-Karlsruhe	948 (44.7%)	1999 (45.9%)	607 (42.4%)	1333 (44.8%)
*Age (mean ± SD)* *(missing = 0)*	58.6 (± 3.9)	58.5 (± 4.0)	68.6 (± 2.9)	68.6 (± 2.9)
*BMI (mean ± SD) (missing = 29)*	25.7 (± 4.5)	25.9 (± 4.8)	26.2 (± 4.3)	26.6 (± 4.7)
*Education*^*a*^* (missing* = *1)*				
Low	1108 (52.2%)	2271 (52.1%)	908 (63.4%)	1905 (64.0%)
Middle	626 (29.5%)	1303 (29.9%)	370 (25.8%)	768 (25.8%)
High	388 (18.3%)	783 (18.0%)	154 (10.8%)	304 (10.2%)
*Living with a partner (missing* = *13)*				
No	529 (25.0%)	1111 (25.5%)	515 (36.0%)	1073 (36.1%)
Yes	1589 (75.0%)	3242 (74.5%)	915 (64.0%)	1902 (63.9%)
*Smoking status (missing* = *3)*				
Never	976 (46.0%)	1868 (42.9%)	891 (62.2%)	1887 (63.4%)
Former	613 (28.9%)	1384 (31.8%)	372 (26.0%)	748 (25.1%)
Current	534 (25.2%)	1104 (25.3%)	169 (11.8%)	340 (11.4%)
*Alcohol consumption (missing* = *7)*				
Abstinent	438 (20.6%)	832 (19.1%)	356 (24.9%)	720 (24.2%)
< 19 g/day	1318 (62.1%)	2815 (64.6%)	892 (62.4%)	1852 (62.3%)
≥ 19 g/day	367 (17.3%)	708 (16.3%)	181 (12.7%)	403 (13.5%)
*Physical activity*^*b*^ *(mean ± SD) (missing = 135)*	50.6 (± 37.1)	51.8 (± 38.1)	49.5 (± 35.4)	51.2 (± 36.6)
*Chronic diseases*^*c*^				
Cardivascular disease^d^	245 (11.5%)	503 (11.5%)	295 (20.6%)	614 (20.6%)
Hypertension	720 (34.0%)	1465 (33.8%)	738 (51.6%)	1474 (49.8%)
Diabetes	127 (6.0%)	220 (5.1%)	176 (12.3%)	293 (9.9%)
Any cancer (excluding breast)	110 (5.2%)	198 (4.5%)	96 (6.7%)	192 (6.4%)

^a^Measured by combining school education and professional education

^b^MET hours/week from sports, cycling and walking from the age of 50 +

^c^Diagnosis between birth and baseline

^d^Including myocardial infarction, heart failure, arrhythmia, angina pectoris, stroke, peripheral vascular disease

CVD prevalence at baseline was twice as high in women aged ≥ 65 years compared to the younger ones (20.6% vs. 11.5%). Diabetes prevalence was higher in cases than in controls and also higher in women aged ≥ 65 than in the younger group (Table 1).

Breast cancer specific characteristics of the cases are shown in Additional file 4: Table S1.

### Mortality by case–control status, CVD and age

The median follow-up time was 16.1 years. In total, 1172 cases (33.0%) and 1401 controls (19.1%) died. A total of 48 women (0.4%) were lost to follow-up. The proportion of deceased women was higher among the elderly than among younger women (Table 2). The most common cause of death among cases was breast cancer, with the proportion of those who died from breast cancer being higher in cases aged < 65 years than in cases aged ≥ 65 years (53.6% vs. 39.9%). The proportion of those who died from CVD was greater in older than in younger cases (19.9% vs. 10.4%). In younger controls, other cancer was the primary cause of death (46.0%) whereas in the older ones, it was CVD (32.3%) (Table 2).

**Table 2:** Vitalstatus and causes of death by age group at recruitment

	< 65 years		≥ 65 years	
	Cases (%) n = 2123	Controls (%) n = 4357	Cases (%) n = 1432	Controls (%) n = 2977
Alive at 30/06/2020	1563 (73.6%)	3844 (88.2%)	807 (56.4%)	2054 (69.0%)
Deceased (total)	548 (25.8%)	489 (11.2%)	624 (43.6%)	912 (30.6%)
Of these: cause of death^a^				
Breast cancer	294 (53.6%)	22 (4.5%)	249 (39.9%)	28 (3.1%)
Other cancer	119 (21.7%)	225 (46.0%)	114 (18.3%)	238 (26.1%)
CVD	57 (10.4%)	90 (18.4%)	124 (19.9%)	295 (32.3%)
Chronic lung disease	13 (2.4%)	28 (5.7%)	27 (4.3%)	55 (6.0%)
Other/unknown	58 (10.6%)	109 (22.3%)	107 (17.1%)	279 (30.6%)
Missing death certificate	7 (1.3%)	15 (3.1%)	3 (0.5%)	17 (1.9%)
Lost to follow-up	12 (0.6%)	24 (0.6%)	1 (0.1%)	11 (0.4%)

^a^Percentages for causes of death refer to numbers of deceased (total) (100%)

**Fig. 1:**
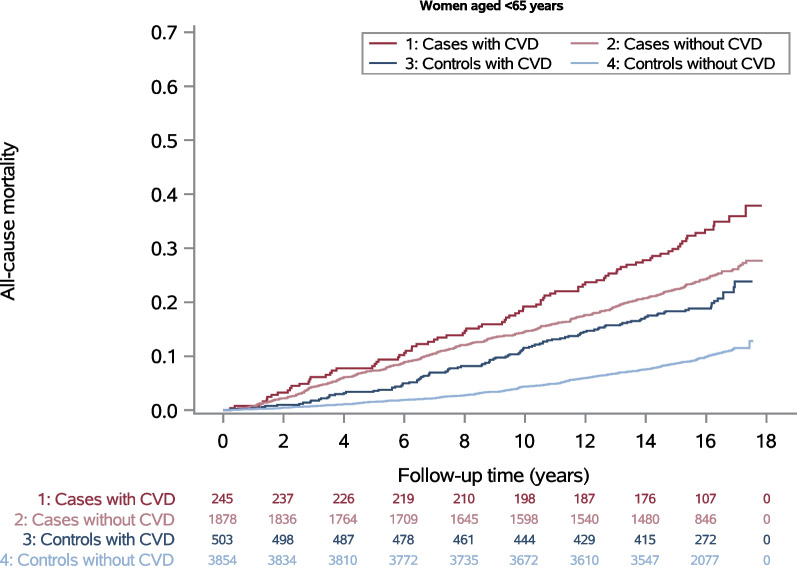
All-cause mortality and numbers at risk for women aged < 65 years

**Fig. 2:**
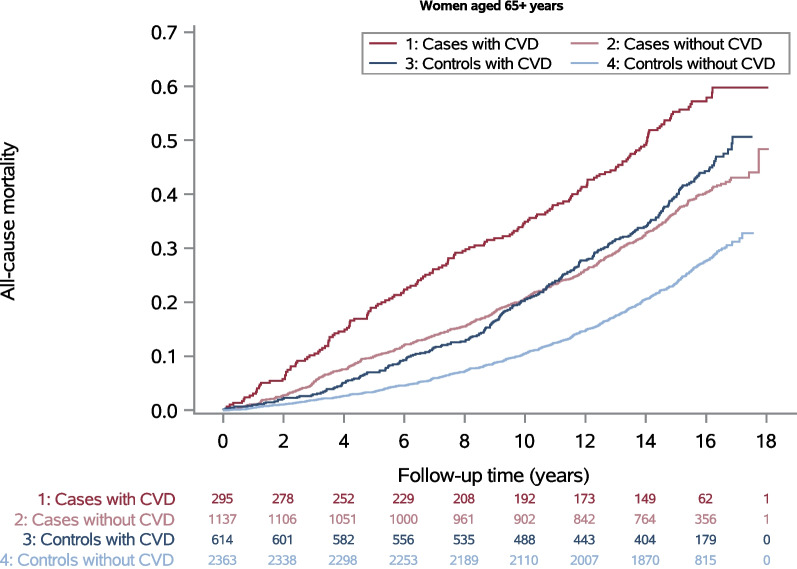
All-cause mortality and numbers at risk for women aged ≥ 65 years

All-cause mortality was higher in cases than in controls and also higher in women with CVD than in women without CVD among both age groups (Figs. 1 and 2). However, in the older age group controls with CVD tended to have a higher mortality than cases without CVD after ten years of follow-up (Fig. 2). For both age groups, the highest all-cause mortality during the entire observation period was found for cases with CVD and the lowest for controls without CVD (Figs. 1 and 2). Mortality from any cancer including breast cancer, was higher in cases than in controls. A relevantly higher cancer mortality in those with CVD compared to those without CVD was found only among cases aged ≥ 65 years at diagnosis.

(Additional file 2: Figure S2 and Additional file 3: S3).

### Association between case-control status, cardiovascular diseases and mortality

CVD at baseline was associated with all-cause mortality in controls < 65 years and in cases and controls ≥ 65 years (Table 3). In the younger age group, there was an interaction between case–control status and CVD (P = 0.020), indicating differential effects of CVD on all-cause mortality in cases and controls. Cases with CVD at baseline had a 1.22-fold higher risk of dying from any cause (95% CI 0.96–1.55) compared to cases without CVD whereas the HR for controls with CVD vs. controls without CVD was 1.79 (95% CI 1.43–2.24). In women ≥ 65 years, the HR for CVD vs. no CVD was 1.44 (95% CI 1.20–1.73) in cases and 1.59 (95% CI 1.37–1.83) in controls (P for interaction = 0.42) (Table 3). We did not find an interaction between cases-control status and CVD for cancer-specific mortality. Point estimates were higher for controls than for cases in women < 65 and vice versa in women ≥ 65 (Table 3). Cases aged ≥ 65 years with CVD had a 1.31 times higher risk of dying from any cancer compared to those without CVD (95%CI 1.02–1.68 for subdistribution HR; cause-specific HR 1.43, 95% CI 1.12–1.82). Subdistribution and cause-specific hazard ratios pointed in the same direction with estimates for cause-specific HRs being slightly higher (Table 3).

**Table 3:** Hazard Ratios and 95% confidence intervals for all-cause mortality and cancer mortality

				< 65 years				≥ 65 years		
Endpoint	Group		HR^a^	95% CI^b^		P^c^	HR^a^	95% CI^b^		P^c^
All-cause mortality	Cases	No CVD	Ref			0.020	Ref			0.42
		CVD	1.22	0.96	1.55		1.44	1.20	1.73	
	Controls	No CVD	Ref				Ref			
		CVD	1.79	1.43	2.24		1.59	1.37	1.83	
Cancer mortality	Cases	No CVD	Ref			0.46	Ref			0.26
		CVD	1.05	0.79	1.42		1.43	1.12	1.82	
	Controls	No CVD	Ref				Ref			
		CVD	1.26	0.88	1.80		1.15	0.86	1.54	

Endpoint	Group		SHR^d^	95% CI^b^		P^c^	SHR^d^	95% CI^b^		P^c^
Cancer mortality	Cases	No CVD	Ref			0.52	Ref			0.27
		CVD	1.02	0.75	1.39		1.31	1.02	1.68	
	Controls	No CVD	Ref				Ref			
		CVD	1.19	0.83	1.71		1.06	0.80	1.42	

Models adjusted for the baseline variables age, BMI, education, living with a partner, smoking status, alcohol consumption, physical activity, diabetes and tumors other than breast cancer. The model includes the interaction term of CVD and case–control status

^a^*HR* Hazard ratio

^b^*CI* Confidence interval

^c^*P* value for interaction between case–control status and CVD

^d^*SHR* Subdistribution hazard ratio

Unadjusted hazard ratios were somewhat larger (Additional file 4; Table S2), indicating the confounding effect of the variables considered in the adjusted model were not large.

Mortality from CVD was higher in cases compared to controls in women < 65 years (cause-specific HR 1.51, 95%CI 1.08–2.13; SHR 1.33, 95%CI 0.95–1.87) but not in those aged ≥ 65 years (Additional file 4: Table S3).

## Discussion

To our knowledge, there are many studies focusing on the risk of death from CVD, but no studies comparing the impact of CVD on all-cause or cancer specific mortality in breast cancer survivors and population controls. Our study showed an effect of CVD at baseline on all-cause mortality in cases and controls after 16 years of follow-up. The relative effect given as hazard ratio appeared to be larger in controls, in particular in women aged < 65 years. The absolute risk difference was increasing over the observation period and in women aged < 65 years it was also larger in controls than in cases. The results therefore do not support the hypothesis that a CVD diagnosis until baseline is a stronger risk factor for total mortality in women diagnosed with breast cancer than in women without this diagnosis. Within the first ten years, cases aged ≥ 65 years without CVD still had a higher mortality than controls with CVD at the same age (Fig. 2). Afterwards, mortality of controls with CVD was little higher than mortality of cases without CVD. An effect of CVD at baseline on cancer mortality was only found for cases aged ≥ 65 years.

When interpreting the results, one has to bear in mind that cases had a higher level of all-cause mortality, and therefore the HR has to be interpreted with caution. Furthermore, when looking at the Kaplan–Meier curves in Figs. 1 and 2, it is noticeable that the risk difference (calculated as difference between the blue, respectively red, curves) was greater in controls aged ≥ 65 than in controls aged < 65 years, whereas the HR for controls aged ≥ 65 was smaller (HR 1.59, 95%CI 1.37–1.83) than for controls aged < 65 years (HR 1.79, 95%CI 1.43–2.24). Even if the difference is small in relative terms, a large absolute difference means that the exposure, in this case CVD, leads to a high number of additional deaths, which could be reduced if the exposure were absent. For our example, this means that the number of additional deaths from CVD is higher in the older controls compared to the younger ones, although this is not reflected in the HR. Therefore, from a public health perspective, it is important to consider both relative and absolute differences.

Furthermore, in controls, the proportion of deaths from CVD among all causes of deaths was higher than in cases (Table 2, 18.4% vs. 10.4% for ages < 65 and 32.4% vs. 19.9% for ages ≥ 65). It is expected that women with known CVD are also at higher risk of dying from CVD and that the presence of CVD has a greater effect on death from CVD than on death from other causes, what is supported by our finding of a higher impact of CVD at baseline on all-cause mortality in controls than in cases.

There are already a number of studies comparing the mortality from CVD in breast cancer cases and controls. The authors of a systematic review of 14 studies on the risk of death from cardiovascular disease and breast cancer summarized that women with breast cancer had a higher CVD mortality than the general population [18]. A nested case–control study published in 2021 reported similar results and found that breast cancer survivors had an increased CVD mortality risk than controls 8 years after diagnosis [17].

In our study, we also found a higher CVD mortality in cases than in controls, but only in women aged < 65 years. Even if breast cancer was the most common cause of death in younger cases, competing risk analysis showed that they also had a higher CVD mortality than controls. We did not observe this for cases ≥ 65 years. This might be explained by adverse events due to cancer treatment. At older ages, CVD is one of the most common causes of death in the general population, so that breast cancer has less impact on CVD mortality than the patients’ age.

Cases and controls had comparable CVD prevalences at baseline, 11.5% in both cases and controls aged < 65 years and 20.6% in both groups aged ≥ 65 years. Self-reported CVD prevalence was relatively high in our study compared to others (12.8% in ≥ 66 US breast cancer patients [11]; 8.5%/18.6% in 60–69/70–79 year old breast cancer patients from the German PASSOS study [23]). Despite the adjustment for baseline CVD prevalence, there was an increased risk of CVD death in younger cases, suggesting that there was an increased incidence of CVD after breast cancer diagnosis in this group, leading to increased CVD mortality.

Two main explanations for the higher CVD mortality in breast cancer patients are given in the literature: firstly, breast cancer and CVD have shared risk factors such as diabetes, resulting in a higher prevalence of these risk factors in women with breast cancer compared to the general population [24]. Secondly, cancer treatments such as radiotherapy can have cardiotoxic effects and cause CVD and cardiovascular death [12, 14].

A register based study by Patnaik et al. including 63,566 women with breast cancer from the U.S. found that CVD at diagnosis had an impact on other cause mortality (HR 1.87, 95%CI 1.80–1.93) as well as on breast cancer mortality (HR 1.24, 95%CI 1.13–1.26). The authors concluded that the reduction of CVD is important in the long-term care of breast cancer patients as it contributes to overall as well as to breast cancer specific mortality [11].

The impact of CVD at diagnosis on mortality, or worse prognosis for women with breast cancer and CVD could be explained by therapy guideline violations in patients with underlying comorbidities [25].

### Strengths and limitations

The data we used for our analyses came from a large population-based case–control study with a median follow-up time of 16.1 years that allows conclusions about long-term effects of CVD. Information on CVD and covariates at baseline were self-reported via standardized face-to-face interviews. A validation study of Kropp et al. showed a good agreement between self-reported hormone therapy data in the MARIE study and information on hormone therapies from attending physicians [26]. It can be assumed that the reliability of data on chronic diseases in MARIE is similarly high.

Furthermore, we accounted for shared risk factors of breast cancer and CVD, including age, obesity/BMI and lifestyle factors such as tobacco use and alcohol consumption.

We only examined the impact of CVD diagnosis between birth and recruitment. Since breast cancer therapies can cause cardiac events and promote the development of CVD [12], we may have missed to demonstrate a differential impact of incident CVD on mortality in the time-course after treatment.

## Conclusion

The highest mortality rates were observed in ≥ 65 years old cases with CVD diagnosis until recruitment. CVD was identified as a risk factor for all-cause mortality of both cases and controls. However CVD at baseline did not appear to be a stronger risk factor in women diagnosed with breast cancer than in women without the diagnosis. CVD was also associated with increased cancer mortality of cases aged ≥ 65 years at recruitment. CVD will become increasingly relevant among the growing and aging group of breast cancer survivors. Since breast cancer therapies may contribute to CVD, attention should be paid to monitoring and preventing CVD and its risk factors in breast cancer patients, especially in older women.

## Supplementary Information

**Additional file 1.** Directed acyclic graph for the association of CVD and all-cause mortality. Green arrow= causal path, red arrows= biasing paths.

**Additional file 2.** Mortality from any cancer for women aged <65 years.

**Additional file 3.** Mortality from any cancer for women aged ≥65 years.

**Additional file 4.** Supplemental Tables 1–3

## Data Availability

The datasets analysed during the current study are not publicly available due to data protection fundamentals but are available from the corresponding author on reasonable request.

## Abbreviations

BMI: Body mass index
CVD: Cardiovascular disease
HR: Hazard ratio
MARIE: Mamma carcinoma risk factor investigation
MET: Metabolic equivalent of task
SHR: Subdistributional hazard ratio
95%CI: 95% Confidence interval
