# Supplementary figures and images for: The impact of cardiovascular disease on all-cause and cancer mortality: results from a 16-year follow-up of a German breast cancer case–control study
Source: Breast Cancer Res. 2023 Jul 27;25:89. doi: 10.1186/s13058-023-01680-x (PMC10373242; doi:10.1186/s13058-023-01680-x)

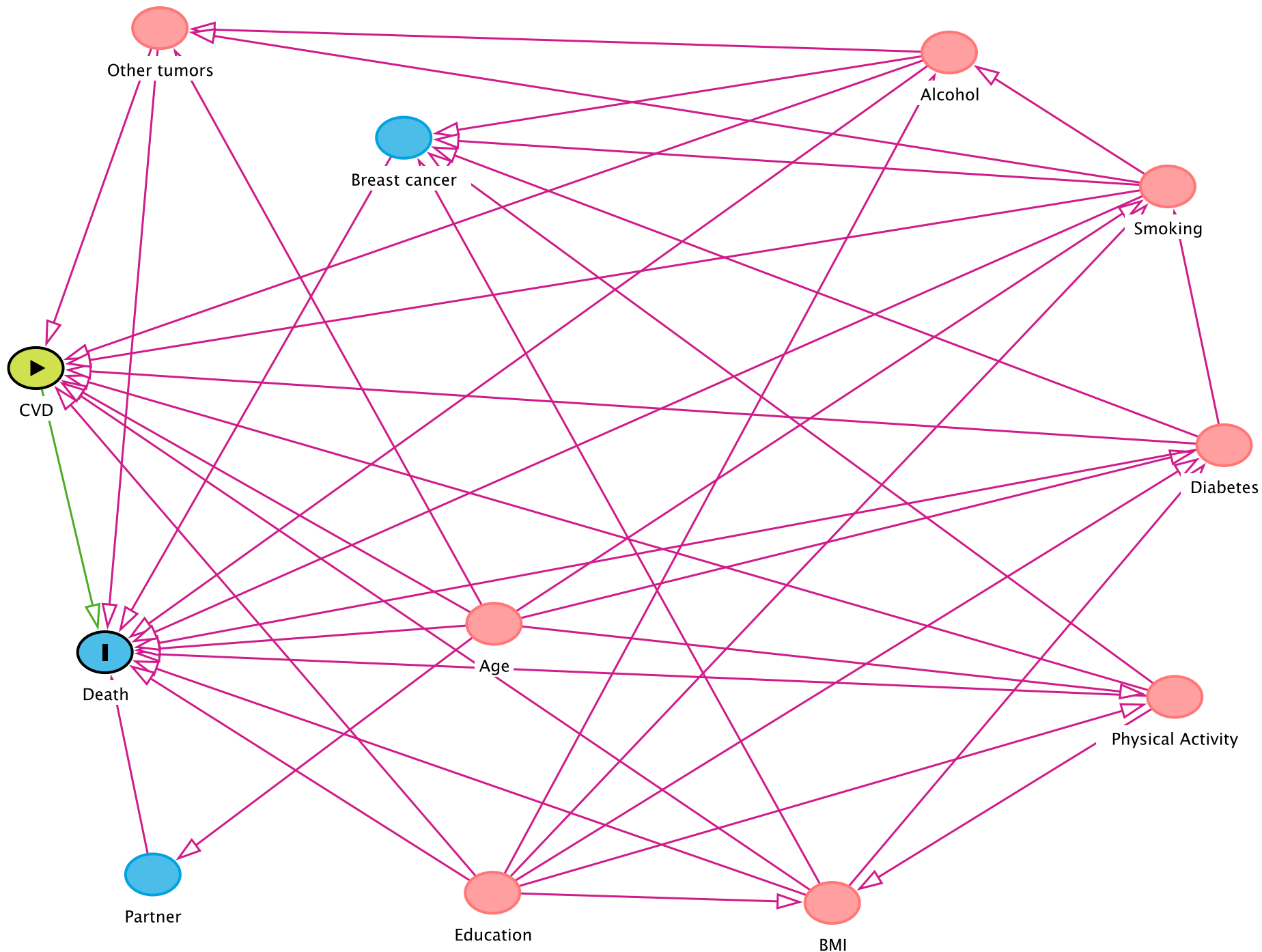

Supplement: Supplementary file 1 — Additional file 1. Directed acyclic graph for the association of CVD and all-cause mortality. Green arrow= causal path, red arrows= biasing paths. [file 13058_2023_1680_MOESM1_ESM.pdf]

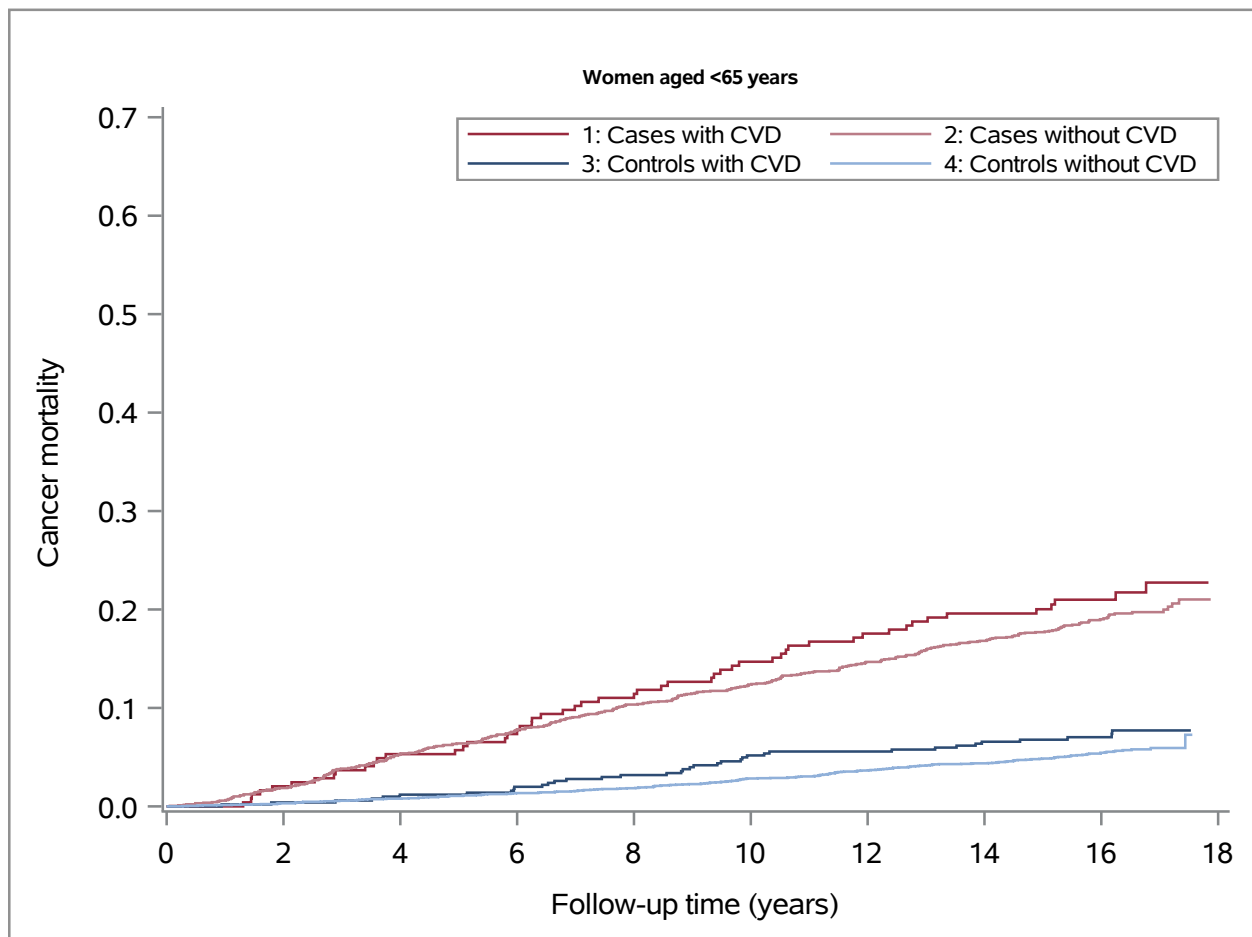

Supplement: Supplementary file 2 — Additional file 2. Mortality from any cancer for women aged <65 years. [file 13058_2023_1680_MOESM2_ESM.pdf]

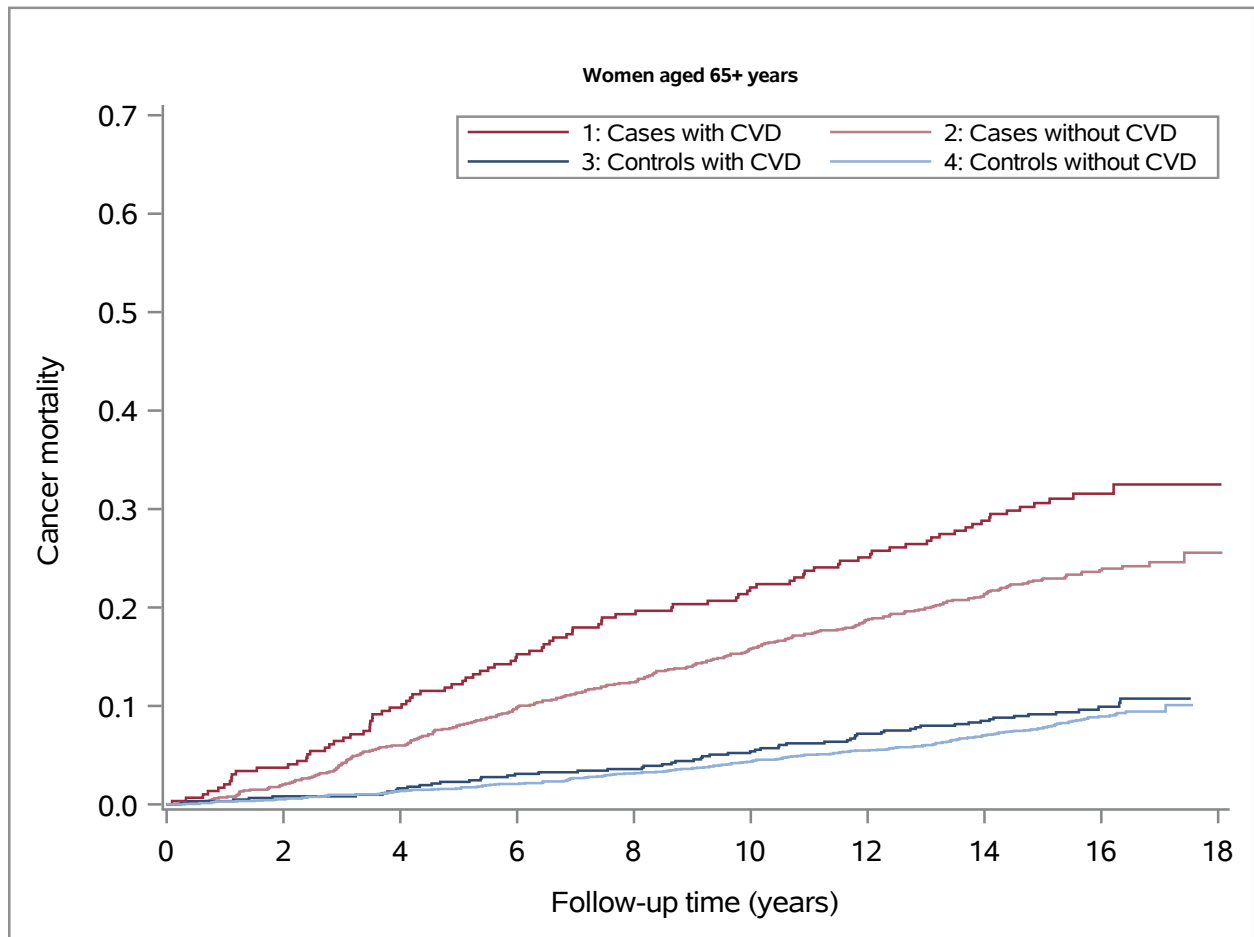

Supplement: Supplementary file 3 — Additional file 3. Mortality from any cancer for women aged ≥65 years. [file 13058_2023_1680_MOESM3_ESM.pdf]
