# Supplementary material for: The impact of cardiovascular disease on all-cause and cancer mortality: results from a 16-year follow-up of a German breast cancer case–control study
Source: Breast Cancer Res. 2023 Jul 27;25:89. doi: 10.1186/s13058-023-01680-x (PMC10373242; doi:10.1186/s13058-023-01680-x)
Supplement: Supplementary file 4 — Additional file 4. Supplemental Tables 1–3 [file 13058_2023_1680_MOESM4_ESM.docx]

Supplemental Files

*Supplemental Table 1: Breast cancer specific characteristics of the MARIE cases*

|  | **Cases** | |
| --- | --- | --- |
|  | **< 65 years (%) n=2123** | **≥ 65 years (%) n=1432** |
| Stage (missing=0) |  |  |
| in situ | 138 (6.5%) | 93 (6.5%) |
| 1 | 962 (45.3%) | 604 (42.2%) |
| 2 | 821 (38.7%) | 569 (39.7%) |
| 3 | 202 (9.5%) | 166 (11.6%) |
| Grade (missing=17) |  |  |
| 1 | 420 (19.9%) | 245 (17.2%) |
| 2 | 1015 (48.1%) | 745 (52.2%) |
| 3 | 537 (25.5%) | 345 (24.2%) |
| in situ | 138 (6.5%) | 93 (6.5%) |
| Hormone receptor status (missing=1) |  |  |
| both pos | 1343 (63.3%) | 949 (66.3%) |
| one pos or hormone therapy | 314 (14.8%) | 215 (15.0%) |
| both neg | 328 (15.4%) | 174 (12.2%) |
| in situ | 138 (6.5%) | 93 (6.5%) |
| Chemotherapy (missing=49) | 1090 (52.1%) | 533 (37.7%) |
| Radiotherapy (missing=41) | 1653 (78.8%) | 1073 (75.8%) |
| Tamoxifen or aromatase inhibitor (missing=171) |  |  |
| no | 402 (19.9%) | 235 (17.2%) |
| either | 1618 (80.1%) | 1129 (82.8%) |

*Supplemental Table 2: Hazard Ratios and 95% confidence intervals for all-cause mortality and cancer mortality*

|  |  |  |  | **<65 years** | |  |  | **≥65 years** | |  |
| --- | --- | --- | --- | --- | --- | --- | --- | --- | --- | --- |
| **Endpoint** | **Group** | | **HR**^a^ | **95% CI**^b^ | | **P**^c^ | **HR**^a^ | **95% CI**^b^ | | **P**^c^ |
| All-cause mortality | Cases | No CVD | Ref. | | | 0.021 | Ref. | | | 0.68 |
|  |  | CVD | 1.41 | 1.11 | 1.77 |  | 1.63 | 1.36 | 1.94 |  |
|  | Controls | No CVD | Ref. | | |  | Ref. | | |  |
|  |  | CVD | 2.04 | 1.64 | 2.54 |  | 1.71 | 1.48 | 1.96 |  |
| Cancer mortality | Cases | No CVD | Ref. | | | 0.42 | Ref. | | | 0.23 |
|  |  | CVD | 1.15 | 0.86 | 1.54 |  | 1.53 | 1.21 | 1.93 |  |
|  | Controls | No CVD | Ref. | | |  | Ref. | | |  |
|  |  | CVD | 1.39 | 0.98 | 1.97 |  | 1.22 | 0.92 | 1.62 |  |
|  |  |  | **SHR**^d^ | **95% CI**^b^ | | **P**^c^ | **SHR**^d^ | **95% CI**^b^ | | **P**^c^ |
| Cancer mortality | Cases | No CVD | Ref. | | | 0.47 | Ref. | | | 0.26 |
|  |  | CVD | 1.13 | 0.84 | 1.50 |  | 1.41 | 1.11 | 1.79 |  |
|  | Controls | No CVD | Ref. | | |  | Ref. | | |  |
|  |  | CVD | 1.33 | 0.94 | 1.89 |  | 1.14 | 0.85 | 1.51 |  |
| ^a^ HR: hazard ratio | |  |  |  |  |  |  |  |  |  |
| ^b^ CI: confidence interval | | |  |  |  |  |  |  |  |  |
| ^c^ P value for interaction between case-control status and CVD | | | | | | | |  |  |  |
| ^d^ SHR: subdistribution hazard ratios | | | |  |  |  |  |  |  |  |

*Supplemental Table 3: Cause-specific and subdistributional Hazard Ratios and 95% confidence intervals for CVD mortality*

|  |  |  | **<65 years** | |  | **≥65 years** | |
| --- | --- | --- | --- | --- | --- | --- | --- |
| **Endpoint** | **Group** | **HR**^a^ | **95% CI**^b^ | | **HR**^a^ | **95% CI**^b^ | |
| CVD | Controls | Ref. | | | Ref. | | |
|  | Cases | 1.51 | 1.08 | 2.13 | 0.97 | 0.79 | 1.21 |
|  |  | **SHR**^c^ | **95% CI**^b^ | | **SHR**^c^ | **95% CI**^b^ | |
| CVD | Controls | Ref. | | | Ref. | | |
|  | Cases | 1.33 | 0.95 | 1.87 | 0.85 | 0.68 | 1.05 |
| Models adjusted for the baseline variables age, BMI, education, living with a partner, smoking status, alcohol consumption, physical activity, CVD at baseline, diabetes and tumors other than breast cancer. | | | | | | | |
| ^a^HR: hazard ratio (cause-specific) | | | |  |  |  |  |
| ^b^CI: confidence interval | | |  |  |  |  |  |
| ^c^SHR: subdistribution hazard ratios | | | | |  |  |  |
